# ECM-binding properties of extracellular vesicles: advanced delivery strategies for therapeutic applications in bone and joint diseases

**DOI:** 10.1186/s12964-025-02156-5

**Published:** 2025-04-02

**Authors:** Peng Wang, Johanna F. A. Husch, Onno J. Arntz, Peter M. van der Kraan, Fons A. J. van de Loo, Jeroen J. J. P. van den Beucken

**Affiliations:** 1 Department of Experimental Rheumatology, Radboud University Medical Center, Nijmegen, The Netherlands; 2 Radboud Institute for Medical Innovations, Nijmegen, the Netherlands; 3 Department of Dentistry - Regenerative Biomaterials, Radboud University Medical Center, Ph v Leijdenln 25, Nijmegen, 6525EX The Netherlands

**Keywords:** Bone, Extracellular vesicles, Extracellular matrix, Delivery system, Joint

## Abstract

Extracellular vesicles (EVs) and the extracellular matrix (ECM) are essential in maintaining bone and joint health by facilitating intercellular communication, regulating tissue processes and providing structural support. EVs with a large surface area carry diverse biomolecules to steer the function of cells in their surroundings. To understand how EVs localize to specific sites, we here review the available knowledge on EV surface biomolecules and their interactions with ECM components that are crucial for regulating bone remodeling, cartilage maintenance, and immune responses, playing roles in both tissue homeostasis and pathological conditions, such as arthritis and osteoporosis. More importantly, using analyses of animal experimental data, we illustrate the effect of ECM-based biomaterials (e.g. hydrogels, decellularized matrices, and ECM-mimetic scaffolds) as carriers for EVs toward effective EV delivery in regenerative and immunomodulatory therapies in bone and joint tissue. These biomaterials enable sustained release and targeted delivery of EVs, promoting bone and cartilage regeneration. The insights of this review can be utilized to advance the development of cutting-edge therapies for skeletal tissue regeneration and disease management.

## Introduction

Bones and joints protect the integrity of skeletal structures with unique yet interconnected functions. Clinicians face substantial clinical challenges in addressing various bone and joint diseases, such as osteoporosis and arthritis [1, 2]. Globally, osteoporosis affects about 6.3% and 21.2% of men and women over 50, respectively. It is a socioeconomic burden due to healthcare expenses and the rising incapacity to work with patient aging. Moreover, bone fractures/defects are more common in osteoporotic patients [3]. Enhancing bone repair is required for faster fracture healing, and improved implant engraftment is currently pursued by injecting bone replacement materials and functionalizing these materials with growth factors. In addition, osteoarthritis (OA) characterized by chronic pain and loss of mobility is the most common form of arthritis in adults. OA is rising because of population growth and ageing, and the lack of an effective cure. The demand on health systems for care of patients with OA, including joint replacements is increasing [4, 5]. The cause of OA remains to be determined, whilst all patients present loss of cartilage as the main characteristic for this disease. Repairing the affected cartilage is difficult, as this tissue is not vascularized and has naturally a much slower turnover. For OA, the use of hydrogels to enhance cartilage repair is under investigation [6].

Osteoclasts, osteoblast, chondrocytes, as well as synovial cells and immune cells are the main cell types involved in bone and joint homeostasis and dysregulation. Besides, the extracellular matrix (ECM) is essential to cellular functions and represents the major acellular component of bones and joints. ECM is a naturally occurring substance that includes biochemical and biophysical components in the direct vicinity of the living cells [7]. Bone ECM is primarily composed of type I collagen, the fibrous protein that provides tensile strength, and a mineral referred to as (carbonated) hydroxyapatite that provides compressive strength. Furthermore, non-collagenous proteins such as osteocalcin, osteopontin and bone sialoprotein, play critical roles in regulating bone ECM remodeling and cell behavior [8]. In addition, ECM in cartilage and synovial tissues involves cell migration on the microscale and load bearing on the macroscale of the joint [9]. In contrast to bone ECM, cartilage ECM is primarily composed of type II collagen and proteoglycans. Proteoglycans are large molecules with a protein core and multiple glycosaminoglycan (GAG) chains, which are negatively charged and provide the cartilage with its characteristic compressive resistance. The synovium envelops the joint cavity, consisting of two unique layers: a lining layer and a fibrous-areolar sub-lining layer. The lining features an intermittent ECM composed of type III collagen along with laminin, which plays a crucial role in controlling joint lubrication and nutrient exchange through the synovial fluid [10]. Consequently, pathological changes of bones and joints will occur in response to atypical alterations in the structure and composition of tissue specific ECM.

Over the past years, explorations on the diagnostic and therapeutic potency of extracellular vesicles (EVs) have emerged for bone and joint research [11, 12]. EVs are lipid bilayer vesicles with broad size ranges from 30–2000 nm. Based on size, EVs are categorized as (endosomal derived) exosomes and microvesicles derived from plasma membrane/cell surface (30–1000 nm), and larger sized (> 1000 nm) apoptotic bodies, as well as large oncosomes [13]. EVs contain cargos comprising proteins, lipids, metabolites, and nucleic acids, and hence have been recognized as an essential means for intercellular communication [14]. For example, the signaling pathways for Wnt/β-catenin and NF-kB are involved in EV-mediated bone metabolism [15, 16]. Various proteins in the EV cargo, including transforming growth factor beta (TGF-β), osteoprotegerin, and EGFR ligand amphiregulin (AREG), contribute to the biological effects of EVs [17–19]. Additionally, EVs derived from infiltrating immune cells and fibroblast-like synoviocytes (FLSs) mediate communication in the deregulation of joints during local inflammation. The TNF-positive EVs derived from FLSs in rheumatoid arthritis (RA) have been confirmed to induce apoptosis resistance in T cells [20]. Furthermore, T cell EVs increased synthesis of matrix metalloproteinase (MMPs) and prostaglandin E_2_ (PGE_2_) by FLSs, causing ECM degradation in cartilage [21]. Through increasing the expression of MMPs‐inhibitor TIMP‐3 and lowering the level of ADAMTS-5 (A Disintegrin and Metalloproteinase with Thrombospondin motifs 5) of articular chondrocytes, our group previously reported that bovine milk EVs carrying TGF-β positively regulate chondrocyte homeostasis and protect against cartilage destruction [22].

### EV luminal loading

It has been widely reported that EVs carry cargos, such as proteins, RNA, and small molecules as therapeutic delivery systems. Two primary approaches have been commonly used: passive loading that cargo is naturally incorporated into the EVs, and active loading, which involves techniques like electroporation to increase loading efficiency [23]. For instance, recombinant proteins such as Yap1 have been loaded into platelet-derived EVs through electroporation to promote tendon stem cell rejuvenation for tendon regeneration [24]. Likewise, CCL2-siRNA has been loaded into neural stem cells to treat traumatic injury in the spinal cord, highlighting the potential for regenerative medicine [25]. In addition, genetically engineered cells have also been used to produce EVs containing specific therapeutic proteins or RNA. For example, long non-coding RNA MEG3 was used in EV-based therapy for osteosarcoma cells. The EVs were isolated from MSCs that have been transfected with MEG3 and these EVs were then used to inhibit osteosarcoma cell growth. In these contexts, EVs serve as highly effective delivery vehicles, protecting the inside cargo from degradation and ensuring targeted delivery to the tissues.

### EV surface interactions

A large number of surface biomolecules have been identified to anchor on the EV surface for molecular interactions [26], attracting much attention from various fields of biomedicine due to some critical functional significances. For instance, autocrine EVs derived from HT1080 human fibrosarcoma cells have been reported to promote efficient and directional parent cell motility by transporting fibronectin and connecting with multiple integrins to form nascent adhesions [27]. In autoimmune diseases, EVs associate with autoantibodies to form pro-inflammatory immune complexes which contribute to disease pathology. Specially, the efficacy of antigen presentation has shown to be increased largely when B cell-derived EVs carry the functional peptide major histocompatibility complex (MHC) [28]. Our previous work demonstrated that the presence of IgM rheumatoid factor on circulating EVs in a subset of rheumatoid arthritis patients reflects changes in pre-B-cell immunity and disease activity more rapidly than changes in circulating levels of “free” IgM rheumatoid factor, which is associated with higher disease activity [29].

### EV-ECM interactions

**Fig. 1:**
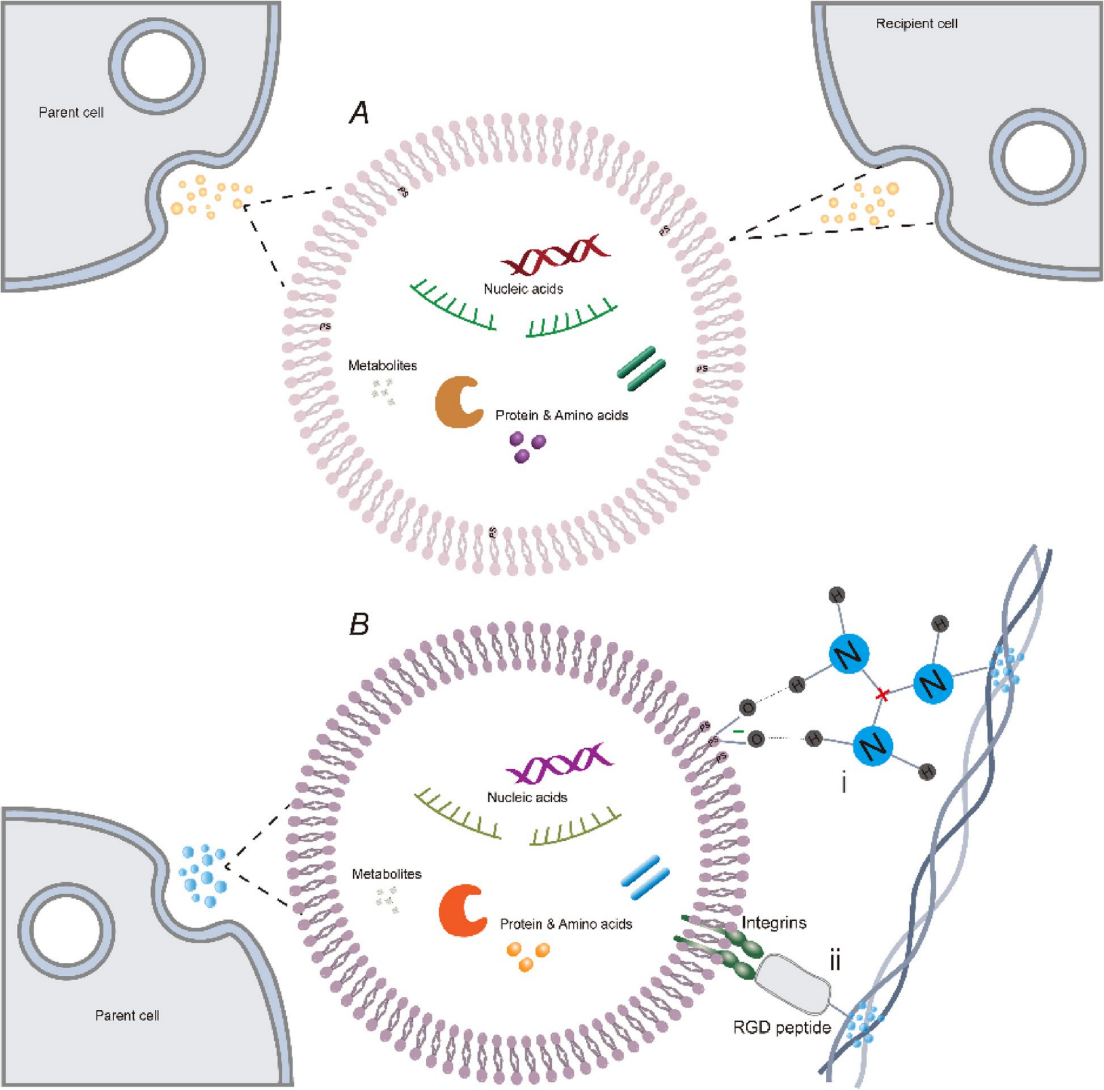
Biomolecular interactions between EV surface and the ECM. Once being released from parent cells, (**A**) one of subpopulation of EVs are “bare EVs” within PS presenting on the inside membrane and directly taken up by cells. (**B**) another subset of EVs engage with the ECM through (i) hydrogen bonds between negatively charged PS on the outer membrane of the EVs and positively charged moieties within ECM proteins; or (ii) incorporating a molecular sequence, such as an RGD peptide that interacts with integrins, allows for the selective capture of specific subpopulations of EVs anchoring ECM. PS: Phosphatidylserine; RGD: amino acids arginine (R), glycine (G), and aspartic acid (D)

The interactions of EV surface molecules with ECM components likely regulate both cellular activities and the physical properties of tissues. Cells receive cues from the ECM, which in turn influences internal signaling that governs the formation of EVs [30]. After being released, a range of biomolecular and physical factors decide if EVs are integrated into the ECM at their site of release to participate in ECM activities either to be taken up by other cells or to be distributed to more distant tissues [31]. Once enveloped in the macromolecular ECM environment, it has been reported that EVs are categorized as an integral ECM component [32] (Fig. 1). On the one hand, EVs interact with ECM such as fibronectin and collagen via hydrogen bonds facilitated by heparin-binding domains rich in basic amino acids. These amino acids comprising positively charged groups form hydrogen bonds with negatively charged phosphates on EV membranes. It has been reported that such bonds exist among EVs derived from cells residing in bone tissue and collagen [33]. On the other hand, some other binding domains such as the arginine (R), glycine (G), and aspartic acid (D) (RGD) or Globular N-terminal link are key factors enabling the diversity in receptors on the membrane of EVs to attach to the ECM components including α4β1 integrin [34] and CD44 [35].

In view of the importance of the interactions between EVs and the ECM, we here review the available knowledge on EV surface biomolecules toward understanding the interaction between EVs and ECM; we will not focus on biophysical EV–ECM network interactions as this has been done in detail in recent excellent review [36]. Furthermore, using analyses of animal experimental data, we illustrate the effect of ECM-based biomaterials encapsulating EVs for efficient EV delivery in regenerative and immunomodulatory therapies in bone and joint tissue pathologies.

## Surface biomolecules target EVs to ECM in bone metabolism

*Integrins* as one of most well-known adhesive transmembrane receptors expressed on the surface of EVs, contribute to EVs’ selective targeting. Integrins consist of an alpha (18 types) and a beta (8 types) subunit that together provide selectivity of the integrin to binding specific ligands [37]. Collagen and laminin are examples of ECM components that contain motifs that are recognized by specific integrins. Typically, α3β1 are involved in binding to laminin, while α2β1 in collagen binding. For example, very recent study observed as MCF10 breast cancer EVs travel through the interstitium the EVs containing integrin α3β1 have the potential to bind to the laminin-rich ECM. This interaction influences the population of EVs entering blood and lymphatic capillaries and further determines the spatial distribution of bound and free MCF10 EVs within the interstitium [38]. In addition, α2β1 integrin mediated EVs derived from myofibroblasts to preferentially bind to type I collagen, promoting collagen crosslinking to regulate the activity of myofibroblasts in wound healing [39].

Other integrins subtype have been reported in bone metabolism. *Wellington *et al*.* reported that during the process of bone and tooth resorption more osteoclast EVs containing αVβ3 recognized to have affinity with hydrolyzed collagen [40], are detected in bone tissue, while more α4β1-positive osteoclast EVs are detected in dentine [41]. Based on this, they speculated it was the subtype of integrins that targeted EVs to specific ECM. Recently, one paper demonstrated that the proteomic analysis of the proteinaceous cargo of MSCs EVs revealed that identified proteins were predominantly associated with EVs. These MSCs EVs carry a variety of integrins and integrin ligands, such as collagen and laminin family molecules, implying the affinity of EVs to the corresponding ECM components in tissues [42].

*Fibronectin 1 (FN1)* is a glycoprotein that interacts with collagen, and proteoglycans made of heparan sulfate. The viscoelastic fibrils assembled by FN1 facilitate the maturation and tissue specificity of the ECM. Therefore, FN1 is often upregulated during embryonic development and wound healing [43]. For instance, the accumulation of the FN1 matrix in wound areas aids in the deposition of collagen and plays a role in the contraction of the wound, and α5β1 integrin as the main receptor on cells to bind FN matrix through Arg-Gly-Asp (RGD) peptide facilitate the attachment of nearby cells, enhancing the stability of the ECM [44]. *Greiling *et al*.* conducted a study showing that in vitro, the movement of fibroblasts from a collagen-rich matrix surrounding a wound to the wound bed relied on FN1. Their findings revealed that without FN1, the migration of fibroblasts into the fibrin clot was reduced by approximately 80%, indicating dependency on FN1 within the provisional matrix of the fibrin clot [45]. In fractures, FN1 functions as an immediate three-dimensional framework post-injury, facilitating the formation of additional ECM components. During the inflammatory phase of healing, neutrophils secrete cellular FN1 as a provisional ECM, setting the stage for the healing process. The interaction of growth factors with FN's III 9-10/12-14 domains plays a role in attracting mesenchymal stem cells (MSCs), fibroblasts, and chondro/osteoprogenitor cells to the injury site [46].

It has been reported that FN1 on the EVs surface was involved in bone defect healing [47]. The researchers intravenously injected labeled injured neuron-derived EVs containing FN1 in rats with a tibial bone or calvarial defect. The EVs went into circulation and migrated to various organs. With optical imaging, they noted an exponential increase in the numbers of EVs in the bone tissues with defects. Moreover, the accumulation of EVs in the tissue of bone injury was reduced by coating FN1 inhibitory peptide, GRGDNP (Gly-Arg-Gly-Asp-Asn-Pro), suggesting FN1 directed EV targeting of bone.

## Surface biomolecules target EVs to ECM in joint dysregulation

CD44, a member of the homing cell adhesion molecules (HCAMs), is involved in recruitment of immune cells and tumor cell metastasis [48, 49], and is regarded as the primary receptor of hyaluronan (HA) through an amino-terminal domain [50]. For instance, in bone metastases, HA retention on the surface of cancer cells is possibly mediated by CD44. *Hiraga *et al*.* reported that cell surface-associated HA decreased in CD44-knockdown cells and increased in CD44-overexpressing cells. Immunohistochemical examinations have shown that HA and CD44 frequently co-localize in bone metastases of certain cancer cells, suggesting that CD44-mediated capture may increase the local concentration of HA in bone metastases [51]. In addition, deposits of HA in the synovial compartment have been linked to RA. Research by *Hayes *et al*.* revealed that synovial tissue from RA patients exhibited a 3.5-fold increase in CD44 expression compared to that from osteoarthritis patients, and a 10.7-fold increase compared to patients with joint trauma but without chronic arthritis, suggesting a significant upregulation of CD44 in the synovial cells of RA patients, with the expression levels of CD44 in synovial tissue being associated with the intensity of inflammation [52]. Moreover, high molecular weight (HMW) HA polymers undergo depolymerization, resulting in the formation of HA fragments, which are hypothesized to activate inflammatory responses from inflammatory cells by signaling through CD44 [53].

Recently, a direct bond between EVs derived from adipose stem cells (ADSCs) and HA matrix has been reported in a model of human osteoarthritic synoviocytes. In this study, ADSC-derived EVs were shown to have increased CD44 level when ADSCs were cultured on HA-coated surfaces and these EVs were preferably involved in the internalization process by fibroblast-like synoviocytes, meaning this interaction between the EVs and HA matrix was crucial for efficient recruitment. This interaction was supposed to be one of the mechanisms involved in superior cartilage regeneration upon intra-articular administration of stem cell EVs in combination with HA, compared to that of HA [54] or EVs alone. However, the increased CD44 level on EVs was limited and inconsistent in this study. To overexpress CD44 on EVs, stable cell lines CD44v6 (CD44 variant isoform 6) overexpression could be established using lentivirus transfection or gateway systems to produce more EVs CD44 [55].

FN1 plays a pivotal role in the development of synovial fibroblasts of RA [56]. This ECM protein, synthesized by synovial fibroblasts, contributes to the composition and structure of type III collagen along with laminin in joint, thereby influencing the architectural integrity of joints. In the context of RA, overexpression of FN1 is linked to synovial membrane thickening and pannus formation, a critical feature of rheumatoid arthritis characterized by immune cell invasion and subsequent cartilage and bone degradation [57].

Coherently, *Skriner *et al*.* reported that the presence of FN1 was distinctively observed in EVs derived from RA patients, in contrast to those from patients with reactive arthritis or osteoarthritis. This underscores that as one marker, FN1's specificity to RA pathophysiology, is particularly in relation to EVs [58]. *Foers *et al*.* reported that FN1 is detectable on human synovial fluid-derived EVs isolated and purified through ultracentrifugation in combination with size exclusion chromatography, as called “dense” or “corona” EVs [59]. Considering the documented pro-inflammatory properties of EV-fibronectin complexes, previous experimental findings might have been influenced by the potential co-isolation artifact of FN1 and EVs [39]. Moreover, in terms of EVs bioactivity and therapeutic roles, *Morteza *et al*.* summarized the interfering effects of protein corona on EVs [60], and FN1 in collaboration with albumin and prothrombin affected distribution capacity and targeted delivery of EVs after introduction in biofluids. They further demonstrated enzymatic treatments such as proteinase K and trypsin that target specific proteins in the corona on the surface of EVs could potentially lead to hydrolysis of those proteins, affecting the structure and function of the corona protein and the EVs themselves. Therefore, it is desirable to functionalize the EVs surface by these enzymes that hydrolyze attached soluble proteins upon EV administration.

## Role of EV surface on ECM remodeling of bone and joint

### ECM degradation in bone

Matrix metalloproteinases (MMPs) are well-known proteases including the metzincin superfamily [61]. They act on cholesterol, surface receptors on cell membranes, as well as nearly all kinds of ECM proteins [62]. It is known that osteoclasts activate proteinases such as MMPs and Cathepsin K, primarily associated with the breakdown of triple-helical type I collagen in bone [63]. Recently, *Lingxin *et al*.* observed that the double knockout of Mmp9/conditional Mmp14 mice exhibited decreased homeostatic bone turnover and were shielded from pathological bone loss [64].

According to *d’Angelo *et al*.,* latent transforming growth factor beta 2 (TGFb-2) was exposed and activated by matrix vesicles (MVs) containing MMP-13. They hypothesized that TGF-β activation further causes pre-osteoblastic cells to invade cartilage and differentiate into osteoblasts, accelerating the progress of OA [65]. Besides, *Tang *et al*.* knocked down the expression of MMP2 in mature osteoblasts-derived EVs and demonstrated its critical role in promoting endothelial cell migration, proliferation, and tube formation [66]. Interestingly, recent work reported on EVs containing tissue inhibitor of metalloproteinases 1 (TIMP1), derived from human urine-derived stem cells (USCs) and their impact on bone health among other aging-related characteristics. Specifically, the intravenous injection of USC-EVs in mouse models significantly enhanced bone quality, notably through delivering TIMP1 directly into the aging tissues to inhibit matrix MMPs, which are implicated in the aging process [67].

### ECM degradation in joint

MMPs, such as MMP-1, MMP-2, and MMP-13, are commonly found in EVs derived from various cell types, including synovial cells, osteoclasts, and fibroblasts in OA and RA. These enzymes are involved in the breakdown of ECM components like collagen and proteoglycans, which are crucial for maintaining cartilage integrity. On the surface of EVs, MMPs actively contribute to ECM turnover by cleaving structural proteins, leading to degradation in joint tissues [68]. EVs expressing MMPs facilitate the modulation of inflammation and cellular invasion, both of which are essential for ECM remodeling in joint diseases. The presence of MMPs on the surface of EVs enables these vesicles to interact with recipient cells, triggering the production of additional matrix-degrading enzymes in these cells. For example, EVs from fibroblast-like synoviocytes (FLSs) in RA are involved in upregulating MMP-13 and ADAMTS-5 expression in chondrocytes, leading to further cartilage degradation and facilitating the pathological remodeling of ECM in the joint [69]. These interactions promote tissue invasion and inflammation, accelerating joint destruction in conditions like RA. Thus, EV-associated MMPs not only contribute directly to ECM degradation but also play a pivotal role in the pathological processes that sustain inflammatory joint diseases. In addition, the formation of new blood vessels is driven by the inflammatory environment in the synovial tissue. As collagen and proteoglycans are degraded by MMPs present on EVs, the breakdown of the ECM leads to the release of pro-angiogenic factors like VEGF (vascular endothelial growth factor) and other cytokines [70]. These factors stimulate endothelial cells to proliferate and form new blood vessels, allowing for the increased delivery of inflammatory cells, nutrients, and immune mediators to the joint and exacerbating the inflammatory cycle and leading to further ECM degradation. Specifically, the presence of EVs carrying MMPs such as MMP-2, MMP-9, and MMP-13 can further enhance the degradation of ECM components and promote angiogenesis (formation of new blood vessels). This creates a vicious cycle of joint inflammation and tissue destruction MMP9 and MMP8 collaborate to activate MMP2 through the influence of reactive oxygen species, contributing to cartilage collagen breakdown and angiogenesis [71].

### ECM mineralization in bone

Collagen type I in bone or collagen type X in endochondral ossification in articular cartilage become calcified due to a large amount of deposited hydroxyapatite crystals intertwined in the collagen triple helix structure [72, 73]. Matrix vesicles (MVs) secreted by osteoblasts, hypertrophic chondrocytes or macrophages are postulated to be the initial site of mineralization in the ECM [74]. Annexin calcium channeling proteins anchor MVs to collagen fibrils and subsequently facilitate an influx of calcium ions. The pre-apatitic mineral phase was then generated through the interaction of calcium with phospholipids (primarily phosphatidylserine) present in the inner membrane of the MVs. [75]. Specially, annexins (i.e. annexins II, IV, and V), alongside phosphatidylserine and tissue non-specific alkaline phosphate (TNAP), are concentrated on the MV surface, facilitating the sequestration of calcium ions necessary for the formation of hydroxyapatite crystals and shifting the ECM from a primarily organic structure to a composite of organic and inorganic materials in bone and endochondral mineralization processes [76, 77].

Furthermore, transforming growth factor beta receptor II interacting protein 1 (TRIP-1) is located in the ER and controls protein synthesis in conjunction with other initiation components [78]. It has been reported that TRIP-1 has an extracellular role as a modulator of matrix mineralization [79]. Recently, EVs derived from MC3T3-E1 cells showed to participate in transportation of TRIP-1 to the ECM, which takes up calcium to precipitate calcium phosphate polymorphs [80], thereby facilitating matrix mineralization.

### ECM synthesis in joint

HA is an anionic and non-sulfated glycosaminoglycan, involved in joint lubrication, cartilage maintenance and longitudinal bone growth [81, 82]. HA is a key element of the cartilage ECM, essential for preserving the health and function of joints. The size of HA molecules plays a significant role in how long they stay within bodily tissues. Specifically, HA with a higher molecular weight tends to stay in the joint or tissue longer before it is degraded and eliminated from the body, thereby extending its beneficial effects. Conversely, HA with a lower molecular weight can more readily move through ECM and interact with specific cells, which have a higher likelihood of inducing immune responses [83]. For example, an imbalance between the production and breakdown of HA in the synovial membrane leads to an accumulation of HA fragments, which intensifies inflammation by triggering immune cells and stimulating the release of inflammatory substances. These HA fragments, alongside CD44, play a role in sustaining ongoing inflammation and contribute to the continuous harm seen in the joints affected by RA [84].

**Fig. 2:**
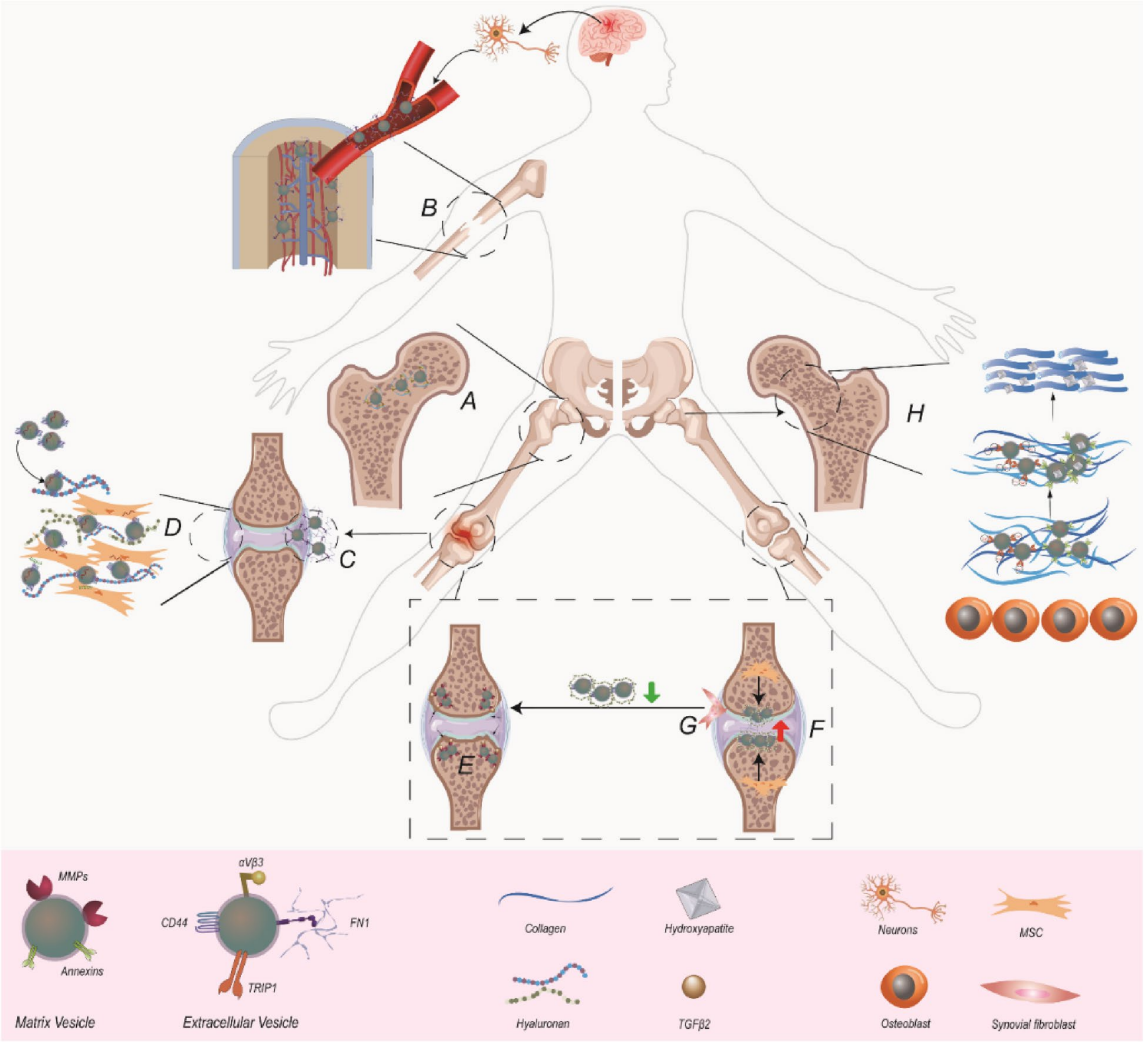
EVs and ECM interaction as modulators in bone and joint homeostasis and dysregulation. **A** αVβ3 osteoclast EVs assemble in the sites of bone resorption. **B** The FN1 around the surface of EVs released by damaged neurons facilitated EV transportation to the site of bone injury via the circulatory system. **C** FN1 on EVs facilitates the attachment of EVs to cells and the internalization or better presentation of associated autoantigens to immune cells in RA. **D** Adipose MSC EVs interact with the HA of inflamed synovial membrane, promoting their accumulation and subsequent release of therapeutic cargos. **E** TGF-β activation by MMP13-MVs potentially induces the differentiation of pre-osteoblastic cells infiltrating the cartilage into osteoblasts, eventually causing the replacement of cartilage with bone tissue. **F** Hydroxyapatite crystals constituted by calcium and phosphate ions grow inside MVs containing annexins or around TRI1-MC3T3E1 derived EVs and align themselves with collagen fibers to mineralize ECM. **G** The HA-coated MSC-derived EVs are responsible for the reorganization and restoration of cartilage through integrating multiple ECM components such as FN1. **H** The decreased of HA-coated synovial fibroblast-derived EVs is implicated for the damage of synovial membrane and cartilage in RA. FN1: Fibronectin; RA: Rheumatoid Arthritis; MSC: Mesenchymal Stem Cell; TGF- β: Transforming Growth Factor Beta; MMP: Matrix metalloproteinases; HA: Hyaluronan; TRI1: Transforming Growth Factor Beta Receptor II Interacting Protein 1

EVs containing HA have shown to participate in ECM remodeling [85]. The amount of diffusive transfer of HA into matrix removed from the cell body may be low, but shedding EVs allow for horizontal transfer: the deposition of HA and other components, and the distribution of signaling molecules even at locations far from the cell of origin [85]. As such, the EVs surrounded by HA contribute to the creation of a regenerative microenvironment that supports stem cells and facilitates the replacement of damaged ECM through integration of other matrix elements [86]. For example, HA of synovial fluid influences the recruitment of stem cells to sites of injury and their differentiation into chondrocytes. It was reported that in OA and healthy synovial fluid, 66% of particles were identified as HA-coated EVs or HA particles, but lower proportion in RA [87]. Of note, recent work focused on the contamination of EV samples with HA and its implications for regenerative applications of corona EVs. *Goncalves *et al*.* demonstrated that low and medium molecular weight HA fragments, which might mimic or interfere with the immunomodulatory effects attributed to EVs, are retained in EV fractions isolated using size-exclusion chromatography and tangential flow filtration [88] (Fig. 2).

## EV encapsuled in ECM-based biomaterials in bone and joint

**Fig. 3:**
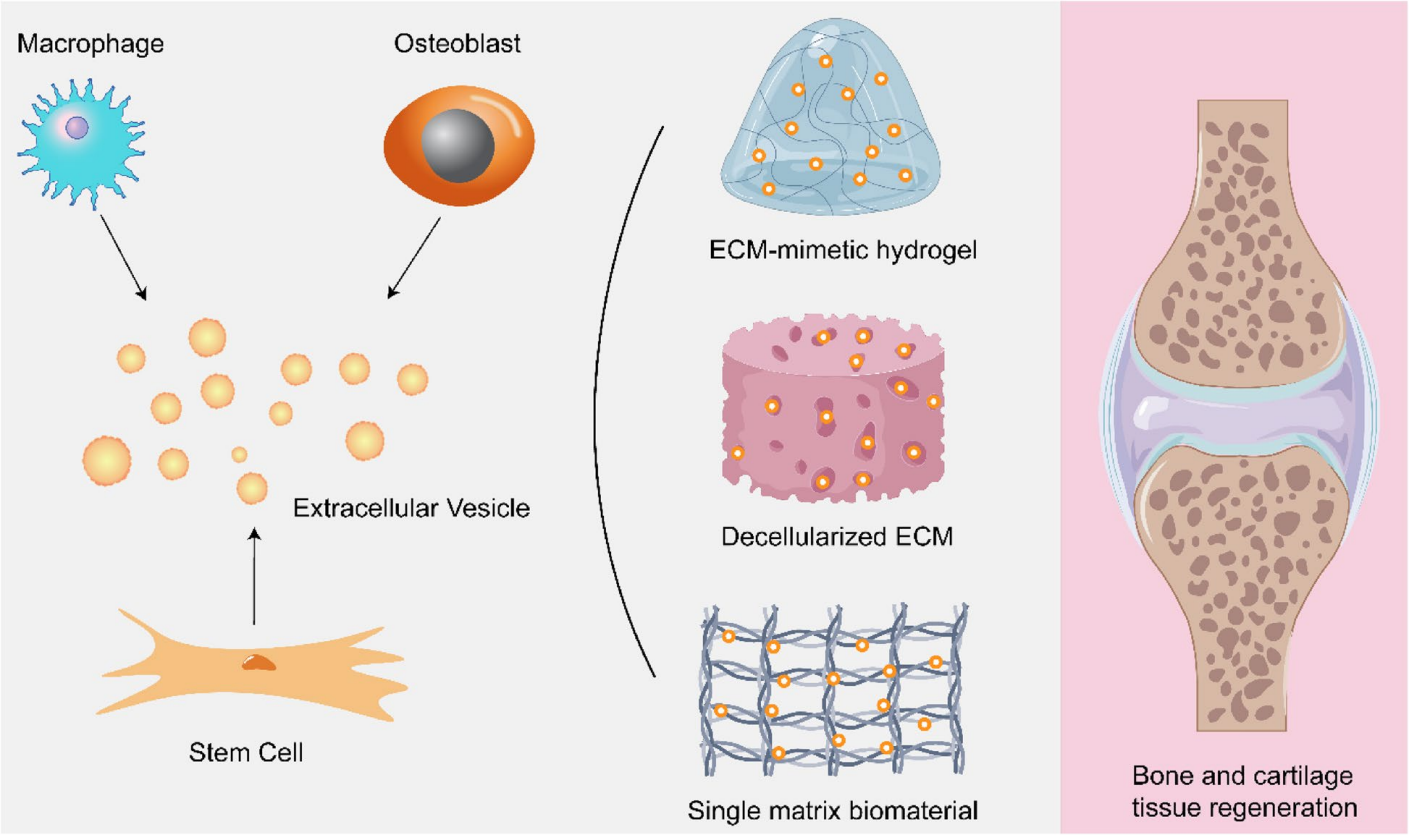
The applications of EVs with ECM-based biomaterials in bone and cartilage regeneration. Once being released from stem cells or osteoblast or macrophages, EVs are loaded into different ECM-based biomaterials such as ECM-mimetic hydrogel, decellularized ECM as well as single matrix biomaterials to realize sustainable local EV delivery. The EV-functionalized ECM scaffolds have been implanted on the at sites of bone or cartilage tissue damage or degeneration to enhanced regeneration and repair

Since MVs were first described as key players in endochondral ossification by Bonucci and Anderson in 1967, multiple reports have focused on the role of EVs derived from MSCs, macrophages, and fibroblasts regarding their contribution to bone and cartilage regeneration [89–91]. Furthermore, EVs have been applied as nanoparticles for systemic administration via intravenous or intraperitoneal injection and oral uptake, leading to systemic dispersion throughout the body. However, it seems straightforward to assume that the therapeutic efficacy of systemic administration is low for local lesions in skeletal tissues. Besides inevitably diluted due to EV clearance, the distant transportation likely makes most EVs fail to contact target tissues/organs and end up in others, such as lungs, liver, and brain [92]. Local injections have been used in preclinical research on therapeutic application in bone and joint disorders, using animal models such as antigen-induced synovitis in pigs and femoral fractures in mice [93, 94]. The results of these studies showed that EVs derived from MSCs have immunomodulatory properties and contribute to tissue regeneration. However, neither the biodistribution nor release kinetics of EVs alone are well regulated. Loaded EVs in or on biomaterials seems a feasible approach to achieve local availability and sustained release toward optimized therapeutic efficacy, especially for bone and cartilage defects, which often require relatively long healing times. Moreover, research on EVs encapsuled in ECM-based biomaterials (e.g. decellularized ECM, specific ECM components, or ECM-mimetic hydrogels) has consequently been investigated for tissue regeneration applications [95] (Fig. 3).

*ECM-mimetic hydrogels* are particularly appealing to EVs due to their customizable mechanical characteristics and various surface modifications [96]. Typically, in a rat femoral fracture model, defect sites were treated with HA hydrogels with embedded EVs from human umbilical cord mesenchymal stem cells (hUMSCs) or human embryonic kidney 293 cells. The findings indicated that the hUMSC-derived EVs facilitated angiogenesis via the activation of HIF-1α-mediated pathways. More importantly, the animals experiment results implied these EVs loaded in HA hydrogel promoted bone regeneration. Since EV biodistribution or release kinetics were not reported, the function of the HA hydrogel as an EV carrier remains unknown [97]. Of note, other work demonstrated that in 14 days an injectable hydroxyapatite combined with HA-alginate hydrogel (i.e. a composite hydrogel) had a relatively slower EV release effect (~ 70%) than pure HA-alginate hydrogel (~ 85%) in vitro. However, it remains unclear which specific molecules contributed to the assembly of EVs on hydroxyapatite, which commonly acts as a ‘magnet’ for multiple biological molecules [98]. Furthermore, the effect of the different released amounts of EVs on bone regeneration is unknown in this study, as the experimental group utilizing pure HA-alginate hydrogel as a carrier of EVs was not included [99]. Recently, an ECM-mimetic hydrogel comprising chitosan-collagen was used to study delivery of murine osteoblast-derived EVs and promote hBMSCs osteogenic differentiation in vitro. This work showed the ratio of chitosan over collagen in hydrogels to play a positive role in immobilizing EVs and subsequent osteogenic effects on hBMSCs [100]. In the transwell system, compared with 8% EV release in 65%Chitosan-35%Collagen hydrogel and 2% in 0%Chitosan-100%Collagen hydrogel, 100% Chitosan-0%Collagen hydrogel had 20% EV release during 7-days observation, with optimal effects on osteogenesis. The researchers speculated that the difference in EV release may be due to the ECM types and EV affinity. Indeed, collagen type I connects with annexins or integrins present on the EV surface, while chitosan absorbs with EVs through electrostatic interactions [101]. Another study demonstrated that a hydrogel device, integrating MSC-EVs within an HA-based hydrogel matrix with specific formulation, has been designed for harnessing the osteogenic and angiogenic potential of the EVs in a controlled-release system, enhancing the regeneration of bone tissue. The study meticulously quantified the release of MSC-derived exosomes from the alginate-HA (ALG-HA) hydrogels. After 14 consecutive days of monitoring, a specific hydrogel ratio of ALG:HA (5%:2.5%) exhibited a slow but steady release of ~ 80% of encapsulated exosomes. In comparison, a different ratio of ALG:HA (5%:5%) showed a release of ~ 60%, and ALG: HA (2.5%:5%) showed the slowest release of ~ 40%. In the animal experiments, the ALG:HA (5%:5%) hydrogel was selected due to its favorable mechanical strength and moderate EVs release profile, and with 50% amount of EVs incorporation, this material had the most optimal effect on bone healing and regeneration [102].

*Zhang *et al*.* recently investigated a specially designed hydrogel combined with EVs as an innovative approach for cartilage repair [103]. Their work introduces the incorporation of EVs derived from BMSCs into a hydrogel composed of alginate-dopamine, chondroitin sulfate, and regenerated silk fibroin (AD/CS/RSF), which exhibits significant adhesive strength to wet cartilage surfaces. Moreover, the hydrogel sustainably released the encapsulated EVs for at least 14 days to ensure a continuous supply of EVs to the cartilage defect site. However, the detailed quantitative data for describing the percentage of EVs released at various time points within the 14-day period was not provided. A recent study reported a composition of EVs and hydrogel made of aldehyde-functionalized chondroitin sulfate and gelatin methacryloyl designed for enhancing ECM synthesis and chondrogenesis. A bicinchoninic acid assay was conducted to quantify the amount of EVs in hydrogel released into phosphate-buffered saline for two weeks, and it showed over 80% of the encapsulated EVs were released by day 14 [104].

### Decellularized ECM

Xenogenic or allogenic bone tissue can be used to prepare decellularized bone ECM scaffolds. These fulfill the requirement for bioactive cellular support, resembling the natural extracellular environment more closely than artificial scaffolds [105]. For the decellularized ECM, bone-derived ECM, particularly decalcified bone matrix, is highly recommended due to its inherent osteoinductive properties that promote bone regeneration [106]. The species of origin should ideally be porcine or bovine ECM due to their biological similarity to humans, which enhances translational relevance [107]. Functionalizing dECM with EVs derived from MSCs is recommended, as EVs have been shown to promote angiogenesis and tissue regeneration. For example, *Xie *et al*.* reported on a bovine decalcified bone matrix coated with fibronectin that was investigated to carry MSC-derived EVs. After EV labeling by carboxyfluorescein succinimidylamino ester, scanning electron microscopy (SEM) images showed that EVs were dispersed evenly in the scaffolds (EV-modified scaffold). In this study, micro-computed tomography scanning and histological analysis showed that more bone formation was observed in EV-modified dECM scaffolds. The Immunohistochemical staining for CD31 proved that vascularization happened in the EV-modified dECM scaffolds, implying enhanced bone regeneration [108]. However, the EV release kinetics from the scaffolds remained unclear in this study. Moreover, one more control group (exogenous EV introduction after in vivo implantation of unmodified scaffold) for functional comparative analysis was lacking.

As for targeting cartilage tissues, cartilage ECM or adipose-derived ECM are desirable to support the differentiation of stem cells into chondrogenic or adipogenic lineages. One recent report demonstrated that the effects of acellular cartilage ECM scaffold (ACECMs) on osteochondral regeneration were enhanced by exogenous Wharton’s jelly MSC-EVs injection. In a rabbit knee osteochondral defect model, an evident longitudinally aligned articular cartilage-like structure was formed at the implantation site. However, only the cytocompatibility of stained BMSCs on the ACECM was observed using scanning electron microscopy (SEM) and confocal laser scanning microscopy; no data on EV retention to the scaffold were described [109]. Similarly, a 3D printed ACECM construct consisting of gelatin methacrylate (GelMA) loaded with MSC-EVs was applied in a rabbit knee osteochondral defect model, potentiating cartilage repair [110]. It showed that this scaffold sustainably retained more EVs both in vitro (for 14 days; > 56%) and in vivo (> 7 days) through nanoparticle tracking analysis (NTA) and IVIS (in vivo imaging system) spectrum, compared to EVs in PBS. Another study observed the cumulative release efficiency of human ADSCs derived EVs from the printed decellularized extracellular matrix (dECM) scaffold was ~ 80% after 24 days, highlighting the hydrogel's capacity for prolonged EV delivery locally at the defect site [111]. In addition to EVs derived from stem cells as the main source of EVs, also other cell types have been used as a source for EVs. Typically, a combination of M2 macrophage-derived exosomes (M2D-Exo) and ACECM scaffold is an innovative approach for osteochondral regeneration, in which M2D-Exo reduced the expression of pro-inflammatory factors and promote M2 polarization, beneficial for cartilage repair [112].

### Single matrix biomaterial

Despite the use of decellularization techniques, isolating and obtaining sufficient tissues remains a time-consuming and costly process, and control over the exact composition of the ECM is limited due to donor variability. Consequently, research has focused on single components of ECM. *Huang & Narayanan* reported on CD63-labeled EVs from human dental pulp stem cells and hBMSCs that were quantified to assess their binding to fibronectin, which was blocked by RGD peptide. Moreover, through determining the connection with collagen I membrane in vitro by ELISA assays, an increased amount of bound EV without saturation was observed. However, the role of collagen membrane on EVs delivery remained unclear after in vivo implantation [113, 114]. Interestingly, one study reported by the same research team demonstrated the prolonged delivery of functionally engineered EVs derived from BMP2 expressing human MSCs has been realized in 4% alginate hydrogels containing RGD peptide in vivo. Since alginate gels are known to exhibit nano-sized mesh networks (normally 10–100 nm) that give alginate gels unique properties to entrap molecules, and the capacity to control the release of these molecules over time. Increasing the concentration of alginate will generally create smaller pores. It is not surprising that compared to 2% alginate, slower EVs release in higher concentration of alginate (4%) has been observed in this study. Furthermore, compared to nearly 50% EV release from 4% alginate hydrogels, similar hydrogels additionally containing RGD peptide showed much lower EV release (20%). Consistently, the use of these hydrogels in a rat calvarial defect model (with 4 and 8 weeks of implantation) showed improved bone regeneration for the 4% alginate hydrogels containing RGD peptide [115]. Similarly, confocal laser-scanning microscopy showed that human periodontal-ligament stem cell (hPDLSCs)-derived EVs, engineered by polyethyleneimine (PEI), had better capacity to adhere onto a commercially available collagen membrane (Evo; name of manufacturer) compared to the membrane without EVs, and promoted better bone regeneration in a calvarium defect animal model. However, the better effect of PEI-EVs on bone regeneration was ascribed to cellular internalization, which was due to the positive charge and proton-sponge property of PEI-EVs. The explanation for more PEI-EVs loaded onto the membrane compared with unmodified EVs was lacking [116] (Table 1).

**Table 1:** Summary of EVs with ECM-based biomaterials in bone and cartilage regeneration

Material	Specific types & source	Application	Results	Reference
ECM-mimetic hydrogel				
Hystem-HP hydrogel	Exosomes derived from hUCMSCs	Rat femoral osteotomy	Enhanced bone healing and angiogenesis for hUCMSCs derived exosomes than HEK293 cell derived exosome or empty control	[97]
HAP/HA-ALG	Exosomes derived from hUCMSCs	Rat cranial defect	1. In 14 days, nearly 70% exosomes release in HAP/HA-ALG hydrogel, and 85% in HA-ALG hydrogel 2. Increased bone healing for exosome + HAP/HA-ALG compared to HAP/HA-ALG or empty control	[99]
Chitosan/Collagen I-b-glycerophosphate	EVs derived from murine osteoblast	Osteogenic differentiation of hBMSCs	1. In 7 days, 20% EV release in 100%Chitosan-Collagen hydrogel, and 8% in 65%Chitosan-35%Collagen hydrogel and 2% in 0%Chitosan-100%Collagen hydrogel 2. In transwell system, the best proliferation and osteogenesis of hBMSCs in 100%Chitosan-Collagen hydrogel + EVs	[100]
Alginate/ HA	Exosomes derived from rat MSCs	Rat calvarial defect model	1. Hydrogel ratio of ALG:HA (5%:2.5%) exhibited a slow but steady release of ~ 80% of encapsulated exosomes. A different ratio of ALG:HA (5%:5%) showed a release of ~ 60%, and ALG: HA (2.5%:5%) had the slowest release rate at ~ 40% 2. Best improvement in bone healing and regeneration in Alginate/ HA + 50% EVs group	[102]
AD/CS/RSF	Exosomes derived from rat BMSCs	Rat osteochondral defect	1. Sustained release of encapsulated exosomes over at least 14 days from AD/CS/RSF hydrogel 2. Improved outcomes in cartilage surface smoothness, continuity, and the generation of hyaline-like cartilage	[103]
Chondroitin sulfate/ gelatin methacryloyl	Exosomes derived from rat BMSCs	Rat distal femoral drill-hole growth plate injury model	1. Over 80% of the encapsulated exosomes were released by day 14 2. Increased chondrocyte anabolism and enhanced growth plate injury repair through ECM remodeling	[104]
Decellularized ECM				
DBM	EVs derived from rat BMSCs	Nude mice subcutaneous	Increased bone induction and blood vessel formation for EVs + rBMSCs compared to other two components alone or scaffold only	[108]
ACECM	Exosomes derived from human umbilical cord Wharton's jelly MSC	Rat and Rabbit femoral trochlear osteochondral defect	Exosomes enhance M2 response compared to PBS Exosomes + ACECM improves osteochondral regeneration compared to ACECM, Exosomes, PBS or sham only	[109]
2% cartilage ECM/GelMA	Exosomes derived from BMSCs	Rabbit patellar groove osteochondral defect	1. In 14 days, nearly 44% exosome release in ECM/GelMA scaffold in vitro In 7 days, better EV retention in 3D printed ECM/GelMA scaffold compared to control (Exosomes in PBS) in subcutaneous mouse model 2. Enhanced M2 response, cartilage and subchondral bone regeneration by ECM/GelMA/Exosomes scaffold compared to no treatment group, GelMA scaffold group, and ECM/GelMA group	[110]
Hydrogel-dECM scaffold	Exosomes derived from hADSCs	Rat knee osteochondral defects	1. Over 80% of the encapsulated exosome were released by day 24 2. The Exosomes-enriched groups showed superior outcomes in terms of the quality of regenerated tissue, including improved matrix deposition, enhanced cellular infiltration, and more robust integration with surrounding tissues	[111]
ACECM	Exosomes derived from rat M2 macrophages	Osteochondral defect model of rats	The promotion of osteochondral regeneration and the regulation of the joint cavity's inflammatory microenvironment	[112]
Single matrix biomaterial				
Type I collagen	Exosomes derived from hDPSCs	Human root slice model in nude mice	Stimulated Exosomes + hDPSCs induced more dental pulp-like tissue regeneration than unstimulated Exosomes + hDPSCs and hDPSCs only	[113]
Type I collagen	Exosomes derived from hMSCs	Nice mice subcutaneous	Increased bone induction and vascularization for stimulated Exosomes + hMSCs than unstimulated Exosomes + hMSCs and hMSCsonly	[114]
Alginate hydrogels containing RGD peptide	EVs derived from BMP2 overexpressing hMSCs	Rat calvarial bone defect	1. In 7 days, 20% EV release in 4%-Alginate-RGD hydrogel, compared with 100% in 2% Alginate hydrogel and 50% in 4% Alginate hydrogel and 2%-Alginate-RGD hydrogel in vitro 2. In 4 and 8 weeks, best bone repair in 4%-alginate-RGD hydrogel hydrogel + EVs, among 4%-alginate hydrogels ± EVs, and alginate-RGD hydrogels only	[115]
Evolution collagen membrane	EVs derived from hPDLSCs (± PEI modification)	Rat calvarial bone injury	Improved bone healing observed for PEI-EVs + hPDLSCs than hPDLSCs or PEI-EVs only	[116]

## Conclusions

The small diameter of EVs leads to large surface-to-volume ratio of EVs [117], allowing for more surface interactions between EV surface structures and molecules within the ECM and on the cell surface. The molecular basis of the interaction between EVs and ECM is expected on the basis of their biochemical composition and chemical bonds. Specifically, hydrogen bonds have been mentioned before to bridge some subsets of EVs and fibronectin or collagen in heparin-binding domain. In addition, other subsets of EVs contain cysteines exposed to the extracellular space, forming covalent bonds with ECM proteins such as laminin [31]. The specificity of these interactions appears important for the regulation of the extracellular microenvironment of bone and joint tissue. In this view, considering the EV surface to interact with a biological environment is of utmost importance to fully exploit the potential of EVs for therapeutic applications. The interactions between EVs and the ECM have been studied for their role in altering the biodistribution of EVs, and considering EVs ability to penetrate tissue barriers, it highlights the growing importance of EV-based therapies.

The synergy between EVs and ECM-based scaffolds is central to the emerging therapeutic strategy. ECM-based biomaterials serve as biologically compatible delivery platforms that closely mimic the native tissue environment. The local delivery of EVs from ECM-based scaffolds is particularly advantageous for bone and joint regeneration. Bone tissue regeneration often requires prolonged therapeutic interventions due to the slow healing process and the need for precise spatiotemporal control over biomolecule delivery. ECM-based scaffolds address this challenge by providing sustained release mechanisms that ensure prolonged EV activity at the site of injury. In cartilage repair, where avascularity and limited cellular turnover present significant obstacles, ECM-based scaffolds embedded with EVs have been shown to enhance chondrocyte activity, protect against ECM degradation, and stimulate cartilage matrix synthesis. Therefore, it should be emphasized that the availability of EVs largely depends on control over EV release. In view of this, cells in the host tissues such as MSCs and endothelial cells are supposed to be recruited on or in the biomaterials [118]. In this review, we summarized that EVs are increasingly being utilized in conjunction with ECM-based biomaterials, such as ECM-mimetic hydrogels and decellularized ECM scaffolds. Moreover, some of these studies explicitly demonstrated the positive role of this hydrogen bond between EVs and ECM-based materials on tissue regeneration, and the essential biomolecules such as integrins and fibronectin have been applied.

### Manufacturing challenges and regulatory hurdles

While the therapeutic promise of EVs delivered via ECM-based scaffolds is clear, several challenges remain to be addressed. Achieving scalable production within low cost and consistent quality control as the regular manufacturing challenges on regenerative medicine. Moreover, the heterogeneity of EV populations poses significant barrier to clinical translation. EV heterogeneity implies that individual vesicles may not possess all the chemical or physical characteristics attributed to the bulk population, indicating that each vesicle might lack certain functional properties including ECM binding ability and related therapeutic functions that are associated with the bulk. For example, Y*ijun Zhou *et al. demonstrated that heparin-chromatography separated the two subpopulations of cancer cell-derived EVs. These two EVs had similar biophysical characteristics but non-heparin binding EVs did not induce ERK1/2 phosphorylation or Ki67 activation, while EVs with heparin binding property induced ERK1/2 phosphorylation and Ki67 activation [119]. Therefore, it is eminent to understand the detailed mechanisms underlying EV-ECM interactions, and optimizing scaffold formulations for specific applications is a critical topic for future research. In addition, the safety, ethical sourcing of both ECM materials and EVs, as well as demonstrating their long-term efficacy in human trials are current regulatory hurdles. By establishing standardized sourcing protocols for ECM materials and EVs, ethical practices, safety, and traceability can be ensured. More importantly, in addition to studying the functional properties of EV-ECM, monitoring for potential chronic toxicity and immune responses should be conducted.

### Key findings and outlook

We summarized the EV surface molecules such as integrins, FN1 and CD44 that guide EVs to interact with ECM components in bone and joint tissues. In addition, MMPs, TRIP-1, and HA contribute to ECM remodeling by modulating key processes such as ECM degradation, mineralization, and synthesis. More importantly, ECM-based biomaterials including hydrogels and decellularized ECM scaffolds provide biologically compatible environments for localized and sustained release of EVs, contributing to bone and cartilage regeneration.

EV-based therapies have shown great promise in treating bone and joint diseases by leveraging combination with ECM-mimetic biomaterials. Future research should focus on optimizing EV-ECM interactions and improving the design of biomaterials to enhance targeted delivery, release control, and therapeutic efficacy. Despite the challenges, such as EV heterogeneity and the complexity of ECM compositions, combining EVs with advanced biomaterials offers a potential pathway for addressing bone and joint conditions like osteoarthritis and bone defects.

## Data Availability

No datasets were generated or analysed during the current study.
